# Chinese Residents’ Perceptions of COVID-19 During the Pandemic: Online Cross-sectional Survey Study

**DOI:** 10.2196/21672

**Published:** 2020-11-25

**Authors:** Tingting Cui, Guoping Yang, Lili Ji, Lin Zhu, Shiqi Zhen, Naiyang Shi, Yan Xu, Hui Jin

**Affiliations:** 1 Department of Epidemiology and Health Statistics School of Public Health Southeast University Nanjing China; 2 Key Laboratory of Environmental Medicine Engineering, Ministry of Education School of Public Health Southeast University Nanjing China; 3 Jiangsu Provincial Center for Disease Control and Prevention Nanjing China

**Keywords:** COVID-19, perception, China, cross-sectional survey, health education, time-varying reproduction number, knowledge, skill, behavior, work resumption, study resumption

## Abstract

**Background:**

COVID-19 has posed a global threat due to substantial morbidity and mortality, and health education strategies need to be adjusted accordingly to prevent a possible epidemic rebound.

**Objective:**

This study aimed to evaluate the perceptions of COVID-19 among individuals coming to, returning to, or living in Jiangsu Province, China, and determine the impact of the pandemic on the perceptions of the public.

**Methods:**

In this study, an online questionnaire was distributed to participants between February 15 and April 21, 2020. The questionnaire comprised items on personal information (eg, sex, age, educational level, and occupation); protection knowledge, skills, and behaviors related to COVID-19; access to COVID-19–related information; and current information needs. Factors influencing the knowledge score, skill score, behavior score, and total score for COVID-19 were evaluated using univariate and multivariate analyses. The time-varying reproduction number (*R_t_*) and its 95% credible interval were calculated and compared with the daily participation number and protection scores.

**Results:**

In total, 52,066 participants were included in the study; their average knowledge score, skill score, behavior score, and total score were 25.58 (SD 4.22), 24.05 (SD 4.02), 31.51 (SD 2.84), and 90.02 (SD 8.87), respectively, and 65.91% (34,315/52,066) had a total protection score above 90 points. For the knowledge and skill sections, correct rates of answers to questions on medical observation days, infectiousness of asymptomatic individuals, cough or sneeze treatment, and precautions were higher than 95%, while those of questions on initial symptoms (32,286/52,066, 62.01%), transmission routes (37,134/52,066, 71.32%), selection of disinfection products (37,390/52,066, 71.81%), and measures of home quarantine (40,037/52,066, 76.90%) were relatively low. For the actual behavior section, 97.93% (50,989/52,066) of participants could wear masks properly when going out. However, 19.76% (10,290/52,066) could not disinfect their homes each week, and 18.42% (9589/52,066) could not distinguish differences in initial symptoms between the common cold and COVID-19. The regression analyses showed that the knowledge score, skill score, behavior score, and total score were influenced by sex, age, educational level, occupation, and place of residence at different degrees (*P*<.001). The government, television shows, and news outlets were the main sources of protection knowledge, and the information released by the government and authoritative medical experts was considered the most reliable. The current information needs included the latest epidemic developments, disease treatment progress, and daily protection knowledge. The *R_t_* in the Jiangsu Province and mainland China dropped below 1, while the global *R_t_* remained at around 1. The maximal information coefficients ranged from 0.76 to 1.00, which indicated that the public’s perceptions were significantly associated with the epidemic.

**Conclusions:**

A high proportion of the participants had sufficient COVID-19 protection knowledge and skills and were able to avoid risky behaviors. Thus, it is necessary to apply different health education measures tailored to work and study resumption for specific populations to improve their self-protection and, ultimately, to prevent a possible rebound of COVID-19.

## Introduction

COVID-19, caused by the novel coronavirus SARS-CoV-2, was first reported in late December 2019 in Wuhan City, China. As a relative of the two conditions caused by previous coronaviruses, namely, the severe acute respiratory syndrome (SARS) and Middle East respiratory syndrome (MERS), COVID-19 has posed a great global threat due to its substantial morbidity and mortality [1,2], and was declared by the World Health Organization (WHO) to be a public health emergency of international concern on January 30, 2020 [3]. Preliminary research has shown that the severity of COVID-19 was lower than that of SARS and MERS; however, it may be more infectious [2,4,5]. According to the WHO’s COVID-19 situation reports [6,7], as of February 15, 2020, a total of 51,857 cases and 1669 deaths were confirmed in only 26 countries, areas, or territories; as of April 21, 2020, the total number of confirmed cases had increased to 2,471,136, with 169,006 deaths, in over 200 countries, areas, or territories. At present, a variety of candidate vaccines have been developed or are undergoing clinical trials to control the pandemic [8,9].

It is essential to pay attention to the public’s knowledge level, attitudes, and perceptions in order to customize the preventive and control measures applied by governments and health authorities during rapidly spreading infectious disease outbreaks [10,11]. During the early period of the COVID-19 outbreak, most participants in the investigations [11-14] conducted in the United States, the United Kingdom, China, and Ethiopia had certain knowledge on COVID-19, such as the main transmission routes and common symptoms, with optimistic attitudes and appropriate practices. However, misconceptions on how to prevent an infection and recommended care-seeking behaviors still existed [11]. Similarly, the knowledge and practices required to combat COVID-19 among high-risk populations are insufficient [14]. Disease perception then plays a relevant role in individuals’ psychological adjustment. The Brief Illness Perception Questionnaire (BIP-Q5) was used to measure the psychological impact during the COVID-19 outbreak in a sample of Spanish adults [15], which showed adequate psychometric properties.

The COVID-19 epidemic in China has been essentially controlled, and the resumption of work and study is proceeding in an orderly manner. Jiangsu Province, located in the eastern part of China, has a large labor import and rich educational resources. Under this scenario, the cross-regional movement of individuals will lead to an increased risk of epidemic imports as well as cluster transmissions of COVID-19. An investigation on the status of protection against COVID-19 among individuals coming to, returning to, or living in Jiangsu Province will help provide information on the current mastery level of knowledge, skills, and protection behaviors; popularize prevention and control knowledge; and tailor health education strategies in a timely manner to ultimately prevent a possible epidemic rebound.

## Methods

### Study Design and Participants

In this study, a cross-sectional online survey was conducted on the public platform created by Jiangsu Provincial People’s Government and managed by the Jiangsu Provincial Center for Disease Control and Prevention (CDC). The platform included Jiangsu health codes, through which every citizen had the obligation to fill in health information during the COVID-19 outbreak; otherwise, they were not permitted to enter/exit public places and their workplaces [16]. The platform included approximately 30,000,000 participants. The questionnaire used in this study has been embedded on the platform since February 15, 2020, participation was anonymous and voluntary, and participants had one chance to fill in their information. This study was approved by the Ethics Committee of the Jiangsu Provincial CDC.

### Data Collection

Data were collected using an online questionnaire through WeChat (Multimedia Appendix 1). The questionnaire was created according to the national guideline for the diagnosis and treatment of COVID-19 and revised via expert evaluation. It includes items on personal information; protection knowledge, skills, and behaviors related to COVID-19; access to information; and current information needs. The Cronbach alpha coefficient and the Kaiser-Meyer-Olkin value for the behavior section was 0.723 and 0.838, respectively, indicating that the research data were relatively true and reliable.

Personal information included demographic data, such as sex, age, educational level, occupation, and place of residence. The knowledge section was composed of 7 single-choice questions and 3 true-or-false questions, which were scored 3 points each, including initial symptoms, distribution of death cases, transmission routes, conditions for killing viruses, mask selection, medical observation days, fever temperature, new coronavirus infection after influenza vaccination, selection of disinfection products, and infectiousness of asymptomatic individuals. The skill section consisted of 9 single-choice questions, scored 3 points each, including cough or sneeze treatment, home quarantine measures, measures from outside to inside (ie, measures implemented by individuals when they go back home from public places or workplaces), mask use, return notice (ie, matters that individuals should pay attention to when they come to or return to Jiangsu Province from other places), washing hands correctly, precautions, quarantine during travel, and attention to household alcohol disinfection. The behavior section comprised 11 scale questions, scored 0-3 points each, including no partying, wearing masks, wearing gloves, washing hands, no contact with live poultry, daily ventilation, weekly disinfection, distinction between the common cold and COVID-19, correct identification of epidemic information, workplace precautions, and community precautions. The highest possible score for each of these sections is 30, 27, and 33 points, respectively. The total score was calculated as follows:

Total protection score = (knowledge score + skill score + behavior score) / 90 × 100.

Three methods were used to ensure data quality. Questionnaires filled out before 12 AM on February 15, 2020, were excluded, as the questionnaire was still in testing and was not officially published. Incomplete questionnaires were also excluded. Finally, questionnaires with irrelevant answers or obvious errors were excluded.

### Statistical Analysis

Frequencies, proportions, arithmetic means, and standard deviations were used to present the data. The chi-square test, the independent samples *t* test (two-tailed), and a one-way analysis of variance were conducted, as appropriate. A multivariate linear regression analysis was performed to identify the factors associated with the knowledge score, skill score, behavior score, and total score for COVID-19. Further, a binary logistic regression analysis was used to explain the selection differences under different characteristics for key items. Unstandardized regression coefficients (β) and odds ratios and their 95% CIs were used to explain associations between variables. The questionnaire data were exported to Microsoft Excel 2016 (Microsoft Corp) for data processing and analysis in combination with SPSS 26.0 (IBM Corp). *P* values of <.05 were considered statistically significant.

In view of the impact of epidemic changes on public perceptions, the time-varying reproduction number (*R_t_*) over a 7-day moving average and its 95% credible interval were estimated in R version 4.0.0 (R Foundation for Statistical Computing) using the method developed by Thompson et al [17], and the serial interval derived from a previous epidemiological survey [18], in combination with the officially published epidemic data of Jiangsu Province, mainland China, and the entire world. Thereafter, the maximal information coefficient [19,20] was applied to test for correlations among the daily participation number, average protection score, number of confirmed cases, and *R_t_*.

## Results

### Participant Characteristics

**Figure 1:**
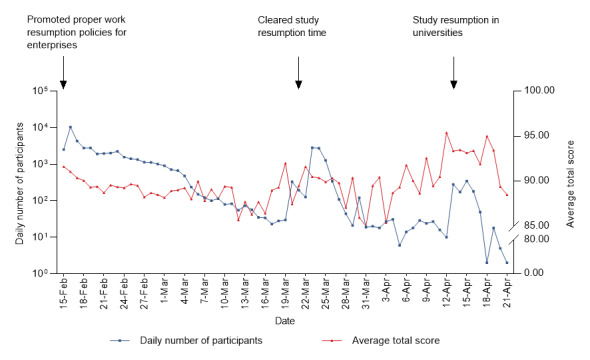
Daily number of participants and average total score.

In total, 52,066 participants were included in the investigation of the status of protection against COVID-19 from February 15 to April 21, 2020, after excluding 344 respondents (including 47 test accounts, 228 with incomplete answers, and 69 with irrelevant answers) (Table 1). Of these, there were 30,212 (58.03%) men, and the male-to-female sex ratio was 1.38:1. The study population mostly comprised those aged 31-40 years (19,131/52,066, 36.74%), followed by those aged 21-30 years (14,226/52,066, 27.32%) and 41-50 years (9885/52,066, 18.99%). In terms of educational level, the proportion of “junior college and bachelor’s degree” was the largest, at 40.89% (21,291/52,066), and the proportion of “master’s degree and above” was the smallest, at only 4.69% (2441/52,066). For the occupational classifications, enterprises (18,187/52,066, 34.93%) and business and service industries (5906/52,066, 11.34%) accounted for a relatively large proportion. One-third of the participants lived in rural areas, while the other two-thirds lived in urban areas. The number of participants involved in the investigation each day is shown in Figure 1, with 3 peaks, which generally corresponded to the time for resuming work and study in batches in Jiangsu Province.

**Table 1:** General characteristics and protection scores of participants.

Characteristic		Participants, n (%)		Total score						Knowledge score							Skill score							Behavior score						
				Mean (SD)		Statistic		*P* value			Mean (SD)		Statistic		*P* value			Mean (SD)		Statistic		*P* value			Mean (SD)		Statistic		*P* value	
**Sex**						*t*=–12.4		<.001					*t*=–10.7		<.001					*t*=–11.2		<.001					*t*=–2.6		.009	
	Male		30,212 (58.03)		89.61 (8.99)							25.41 (4.23)							23.88 (4.14)							31.48 (2.89)				
	Female		21,854 (41.97)		90.58 (8.66)							25.81 (4.19)							24.27 (3.84)							31.54 (2.78)				
**Age (years)**						*F*=387.1		<.001					*F*=245.0		<.001					*F*=381.2		<.001					*F*=34.7		<.001	
	≤20		5432 (10.43)		85.53 (11.74)							23.92 (5.42)							21.98 (5.30)							31.21 (3.44)				
	21-30		14,226 (27.32)		90.76 (7.85)							25.99 (3.79)							24.42 (3.60)							31.38 (2.78)				
	31-40		19,131 (36.74)		90.92 (7.90)							25.88 (3.93)							24.40 (3.61)							31.66 (2.63)				
	41-50		9885 (18.99)		90.25 (8.71)							25.54 (4.18)							24.19 (3.89)							31.59 (2.80)				
	51-60		3031 (5.82)		88.77 (10.13)							25.03 (4.51)							23.51 (4.58)							31.46 (3.05)				
	≥61		361 (0.69)		85.14 (13.60)							23.65 (5.60)							22.34 (5.61)							30.79 (4.43)				
**Educational level**						*F*=1503.8		<.001					*F*=1320.0		<.001					*F*=1240.0		<.001					*F*=27.1		<.001	
	≤Junior high school		14,954 (28.72)		86.57 (10.54)							24.09 (4.90)							22.62 (4.82)							31.34 (3.27)				
	High school and technical secondary school		13,380 (25.70)		89.51 (8.76)							25.22 (4.15)							23.83 (4.05)							31.62 (2.80)				
	Junior college and bachelor’s degree		21,291 (40.89)		92.45 (6.62)							26.67 (3.34)							25.06 (2.99)							31.55 (2.55)				
	≥Master’s degree		2441 (4.69)		92.83 (7.80)							27.06 (3.70)							25.12 (3.37)							31.42 (2.64)				
**Occupation**						*F*=282.4		<.001					*F*=233.6		<.001					*F*=224.0		<.001					*F*=19.4		<.001	
	Government agency and institution		3579 (6.87)		92.57 (7.66)							26.79 (3.57)							24.96 (3.42)							31.65 (2.56)				
	Medical practitioner		2674 (5.14)		93.98 (7.36)							27.42 (3.35)							25.31 (3.26)							31.92 (2.57)				
	Enterprise		18,187 (34.93)		91.26 (7.51)							26.11 (3.70)							24.63 (3.43)							31.49 (2.69)				
	Business and service industry		5906 (11.34)		90.12 (8.08)							25.45 (4.00)							24.14 (3.74)							31.64 (2.67)				
	Farmer^a^		2305 (4.43)		87.34 (10.44)							24.37 (4.81)							23.04 (4.69)							31.33 (3.30)				
	Student		5507 (10.58)		87.13 (11.01)							24.52 (5.17)							22.67 (4.93)							31.35 (3.21)				
	Freelancer		6282 (12.07)		88.62 (9.14)							24.85 (4.37)							23.47 (4.32)							31.57 (2.82)				
	Retiree		420 (0.81)		86.82 (11.36)							24.20 (5.02)							22.71 (4.96)							31.36 (3.02)				
	Unemployed		1398 (2.69)		87.43 (10.37)							24.71 (4.68)							23.20 (4.44)							30.91 (3.52)				
	Other		5808 (11.16)		88.80 (9.18)							25.03 (4.36)							23.58 (4.20)							31.43 (2.99)				
**Place of residence**						*t*=30.0		<.001					*t*=28.5		<.001					*t*=25.0		<.001					*t*=6.3		<.001	
	Urban area		34,426 (66.12)		90.90 (8.19)							25.97 (3.96)							24.37 (3.75)							31.56 (2.68)				
	Rural area		17,640 (33.88)		88.31 (9.84)							24.81 (4.59)							23.40 (4.43)							31.39 (3.14)				
Total		52,066 (100.00)		90.02 (8.87)							25.58 (4.22)							24.05 (4.02)							31.51 (2.84)					

^a^“Farmer” includes agriculture, forestry, animal husbandry, sideline occupations, and fishery.

### Protection Scores

For the total protection score, 65.91% of participants (34,315/52,066) had scores over 90 points. The univariate analysis showed that there were significant differences in the knowledge score, skill score, behavior score, and total score for sex, age, educational level, occupation, and place of residence (all *P*s<.001, except for sex and behavior score [*P*=.009]; Table 1).

#### Partial Score

**Figure 2:**
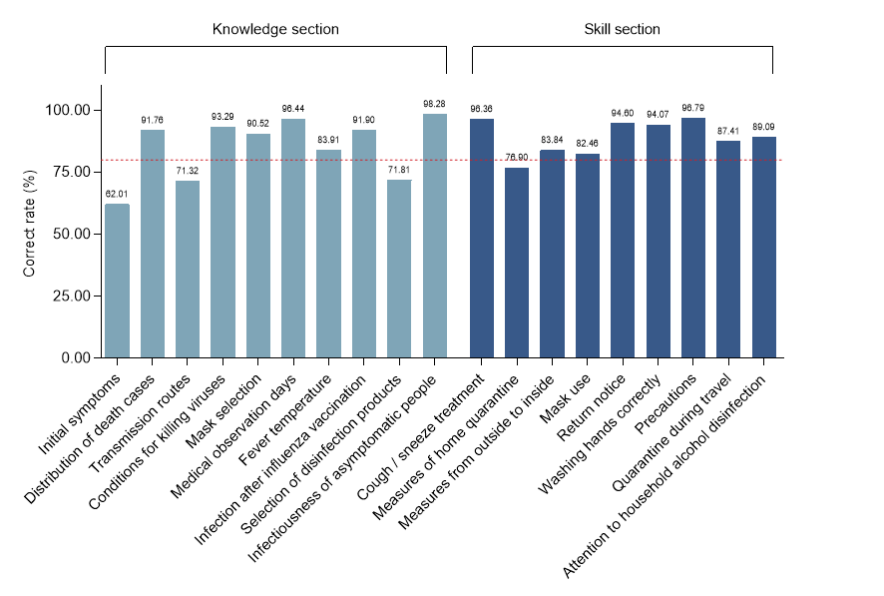
Rates of correct answers related to the knowledge and skill sections of the questionnaire. Reference line: 80.00%, shown in red.

The protection score consisted of three parts: knowledge score, skill score, and behavior score. Initially, we analyzed the first two parts with the same scoring standard, with average scores of 25.58 (SD 4.22) and 24.05 (SD 4.02) (Table 1) and a range of correct answer rates of 62.01%-98.28% (Figure 2). The multivariate linear regression analysis indicated that women; those aged 21-60 years; those with an educational level of high school or greater; those with occupations categorized as government agency and institution, enterprise, business and service industry, medical practitioners, and students; and those living in urban areas had significantly higher knowledge scores than men, those aged ≤20 years, those with an educational level of junior high school or less, those who were unemployed, and those who lived in rural areas (*P*<.001 or *P*=.007; Table S1 in Multimedia Appendix 2). Other than the above variables, those aged ≥61 years (*P*=.003), farmers (*P*=.01), and freelancers (*P*=.04) had significantly higher skill scores (Table S1 in Multimedia Appendix 2).

More concretely, the rates of correct answers for questions on medical observation days, infectiousness of asymptomatic individuals, cough or sneeze treatment, and precautions were higher than 95% in these two sections. Conversely, those of questions on initial symptoms (32,286/52,066, 62.01%), transmission routes (37,134/52,066, 71.32%), selection of disinfection products (37,390/52,066, 71.81%), and measures of home quarantine (40,037/52,066, 76.90%) were relatively low (Figure 2). There were significant differences in the answers to these four questions among the different sexes, age groups, educational levels, occupations, and places of residence (*P*<.001; Table S2 in Multimedia Appendix 2). The binary logistic regression analysis showed that the correct answer rates among women in relation to initial symptoms, transmission routes, selection of disinfection products, and measures of home quarantine were higher than those among men (*P*<.001; Table S3 in Multimedia Appendix 2). Those aged 21-40 years were more aware of disinfection products and measures of home quarantine than those aged ≤20 years (*P*<.001), while those aged ≥51 years were less aware of initial symptoms and transmission routes (*P*<.001 or *P*=.02). The correct rates among the participants with an educational level of high school or greater for these 4 questions were higher than those with an educational level of junior high school and below (*P*<.001). Those with occupations categorized under government agency and institution and medical practitioners were more aware of the initial symptoms, transmission routes, disinfection products, and measures of home quarantine than those who were unemployed (*P*≤.001 or *P*=.002); in particular, medical practitioners had the highest correct answer rates. The correct answer rates for selection of disinfection products and home quarantine measures were higher among those with occupations categorized under enterprise (*P*=.02 or *P*=.001). Students had greater accuracy for initial symptoms, selection of disinfection products, and home quarantine measures (*P*<.001). Those living in urban areas had a higher accuracy for transmission routes and selection of disinfection products (*P*<.001 or *P*=.01; Table S3 in Multimedia Appendix 2).

Thereafter, the actual degree of protection among the participants was examined, and the average score was 31.51 (SD 2.84) (Tables 1 and 2). The multivariate linear regression analysis revealed that women (*P*=.01; Table S1 in Multimedia Appendix 2), those aged 21-60 years (*P*<.001), those with an educational level of high school and technical secondary school (*P*<.001) and junior college and bachelor’s degree (*P*=.046), those with employment (*P*<.001), and those living in urban areas (*P*<.001) had significantly higher behavior scores than men, those aged ≤20 years, those with an educational level of junior high school or below, those who were unemployed, and those who lived in rural areas.

**Table 2:** Protection behaviors and the degree to which participants were able to implement these behaviors (as indicated by the 3-point response “able to”).

Behavior	Participants, n (%)
No partying	48,955 (94.02)
Wearing masks	50,989 (97.93)
Wearing gloves	46,607 (89.52)
Washing hands	49,607 (95.28)
No contact with live poultry	50,191 (96.40)
Daily ventilation	50,670 (97.32)
Weekly disinfection	41,776 (80.24)
Distinction between the common cold and COVID-19	42,477 (81.58)
Correct identification of epidemic information	50,908 (97.78)
Workplace precautions	46,800 (89.89)
Community precautions	47,009 (90.29)

Specifically, a higher proportion of participants were able to avoid gatherings, wear gloves, wash hands in a timely manner, keep away from live poultry and livestock, ventilate each day, and identify information related to the epidemic correctly and believed that precautions in workplaces or communities were in place. For example, 97.93% (50,989/52,066) of participants could wear masks properly when they went out (Table 2). However, 19.76% (10,290/52,066) still could not disinfect their homes each week, which was significantly associated with age, educational level, occupation, and place of residence (*P*<.001; Table S2 in Multimedia Appendix 2). Similarly, 18.42% (9589/52,066) could not distinguish the initial symptoms of the common cold and COVID-19, and this was significantly related to sex, age, educational level, occupation, and place of residence (*P*<.001; Table S2 in Multimedia Appendix 2). The binary logistic regression analysis indicated that compared with participants aged ≤20 years, those aged 31-60 years could disinfect their homes weekly (*P*<.001; Table S3 in Multimedia Appendix 2), and those aged 41-50 years were aware of initial symptom differences between the common cold and COVID-19 (*P*=.04). Those with a high educational level were unable to disinfect their homes weekly and clearly distinguish between the common cold and COVID-19 (*P*<.001 or *P*=.02). In addition to the retirees (*P*=.08), those with the other indicated occupations were able to disinfect their homes weekly (*P*<.001 or *P*=.003). With the exception of those with occupations categorized under enterprise (*P*=.06), people in the other profession categories could distinguish between the common cold and COVID-19 (all *P*s<.05). Participants living in urban areas were more often able to disinfect their homes weekly than those living in rural areas (*P*=.04; Table S3 in Multimedia Appendix 2).

#### Total Score

The total score was obtained by summing up the scores of the three abovementioned parts and converted to the hundred-mark system. The average total protection score was 90.02 (SD 8.87), rising with fluctuations over time (Figure 1), with the highest score (mean 93.98, SD 7.36) observed among medical practitioners (Table 1). The five demographic characteristics (ie, sex, age, educational level, occupation, and place of residence) significant in the univariate analyses (*P*<.001; Table 1) were included in the multivariate linear regression analysis (*F*_19,52046_=343.426, *P*<.001). This showed that women, those aged 21-60 years, those with an educational level of high school or above, those with occupations other than being a retiree, and those living in urban areas had significantly higher total protection scores than men, those aged ≤20 years, those with an educational level of junior high school and below, those who were unemployed, and those who lived in rural areas (*P*<.001; Table 3).

**Table 3:** Results of the multivariate linear regression analysis on factors influencing the total protection score.

Variable			Coefficient		SE		95% CI			*t* test			*P* value			Collinearity statistics (VIF^a^)	
Constant			79.914		0.311		79.305 to 80.523			257.238			<.001			N/A^b^	
**Sex (reference: male)**																	
	Female	0.971		0.077		0.820 to 1.123			12.576			<.001			1.082		
**Age (years; reference:** **≤** **20)**																	
	21-30	4.592		0.221		4.159 to 5.026			20.778			<.001			7.224		
	31-40	5.481		0.226		5.039 to 5.924			24.282			<.001			8.821		
	41-50	4.963		0.232		4.508 to 5.419			21.363			<.001			6.183		
	51-60	3.252		0.267		2.728 to 3.776			12.164			<.001			2.919		
	≥61	0.199		0.535		–0.849 to 1.248			0.373			.71			1.467		
**Educational level (reference: ≤junior high school)**																	
	High school and technical secondary school	2.200		0.103		1.997 to 2.402			21.312			<.001			1.515		
	Junior college and bachelor’s degree	4.206		0.105		4.000 to 4.412			40.056			<.001			1.985		
	≥Master’s degree	4.312		0.197		3.925 to 4.698			21.851			<.001			1.296		
**Occupation (reference: unemployed)**																	
	Government agency and institution	2.712		0.272		2.178 to 3.245			9.964			<.001			3.531		
	Medical practitioner	4.567		0.281		4.016 to 5.119			16.232			<.001			2.872		
	Enterprise	2.272		0.237		1.807 to 2.737			9.580			<.001			9.525		
	Business and service industry	1.762		0.251		1.271 to 2.254			7.027			<.001			4.710		
	Farmer^c^	1.164		0.287		0.601 to 1.727			4.055			<.001			2.598		
	Student	3.830		0.304		3.235 to 4.426			12.609			<.001			6.500		
	Freelancer	1.078		0.248		0.591 to 1.565			4.338			<.001			4.879		
	Retiree	0.647		0.513		–0.359 to 1.653			1.261			.21			1.569		
	Other	0.993		0.250		0.503 to 1.483			3.970			<.001			4.613		
**Place of residence (reference: rural area)**																	
	Urban area	1.046		0.083		0.884 to 1.209			12.623			<.001			1.146		

^a^VIF: variance inflation factor.

^b^N/A: not applicable.

^c^“Farmer” included agriculture, forestry, animal husbandry, sideline occupations, and fishery.

### Information Acquisition and Information Needs

In this study, access to personal protection knowledge on COVID-19 and information needs were also investigated. Television shows, government websites, and news outlets (46,145/52,066, 88.63%), as well as the government’s WeChat public account (45,657/52,066, 87.69%), were the main sources for acquiring personal protection knowledge. The sources for participants of different sexes and places of residence were similar. Participants of all ages rarely obtained protection information from microblogs or via communication among family, relatives, and friends. Those with an educational level of high school or less obtained information mostly from television shows, government websites, and news outlets, while those with an educational level of junior college or above obtained information from the government’s WeChat public account. Participants with occupations categorized under government agency, institution, and enterprise, as well as medical practitioners, obtained information more often from the government’s WeChat public account. Participants believed that the government’s media and WeChat public accounts (48,307/52,066, 92.78%) and authoritative medical experts (46,062/52,066, 88.47%) were the most reliable information sources (Table S4 in Multimedia Appendix 2).

The current information needs of the participants included the latest epidemic developments (46,729/52,066, 89.75%), disease treatment progress (42,181/52,066, 81.01%), and daily protection knowledge (41,451/52,066, 79.61%). Participants of different sexes had large differences in information needs in terms of disease treatment progress, prevention and control status in epidemic areas, and social dynamics; the differences in the other aspects were smaller. Participants of different ages, especially those aged 21-60 years, were very eager to understand the latest epidemic developments. The information needs of those with an educational level of junior high school or below concerned the latest epidemic developments and daily protection knowledge; conversely, the information needs of those with an educational level of high school or above were the latest epidemic developments and disease treatment progress. All participants paid less attention to material supply and social dynamics. Those with occupations categorized under government agency and institution, enterprise, business and service industry, freelancers, medical practitioners, and those who were unemployed had higher needs for epidemic developments and disease treatment progress, while farmers, students, and retirees had higher needs for epidemic developments and protection knowledge. Conversely, those living in rural areas were more interested in obtaining information on epidemic developments and daily protection knowledge than those living in urban areas (Table S5 in Multimedia Appendix 2).

### Correlation Among the Daily Participation Number, Average Protection Score, Number of Confirmed Cases, and R_t_

**Figure 3:**
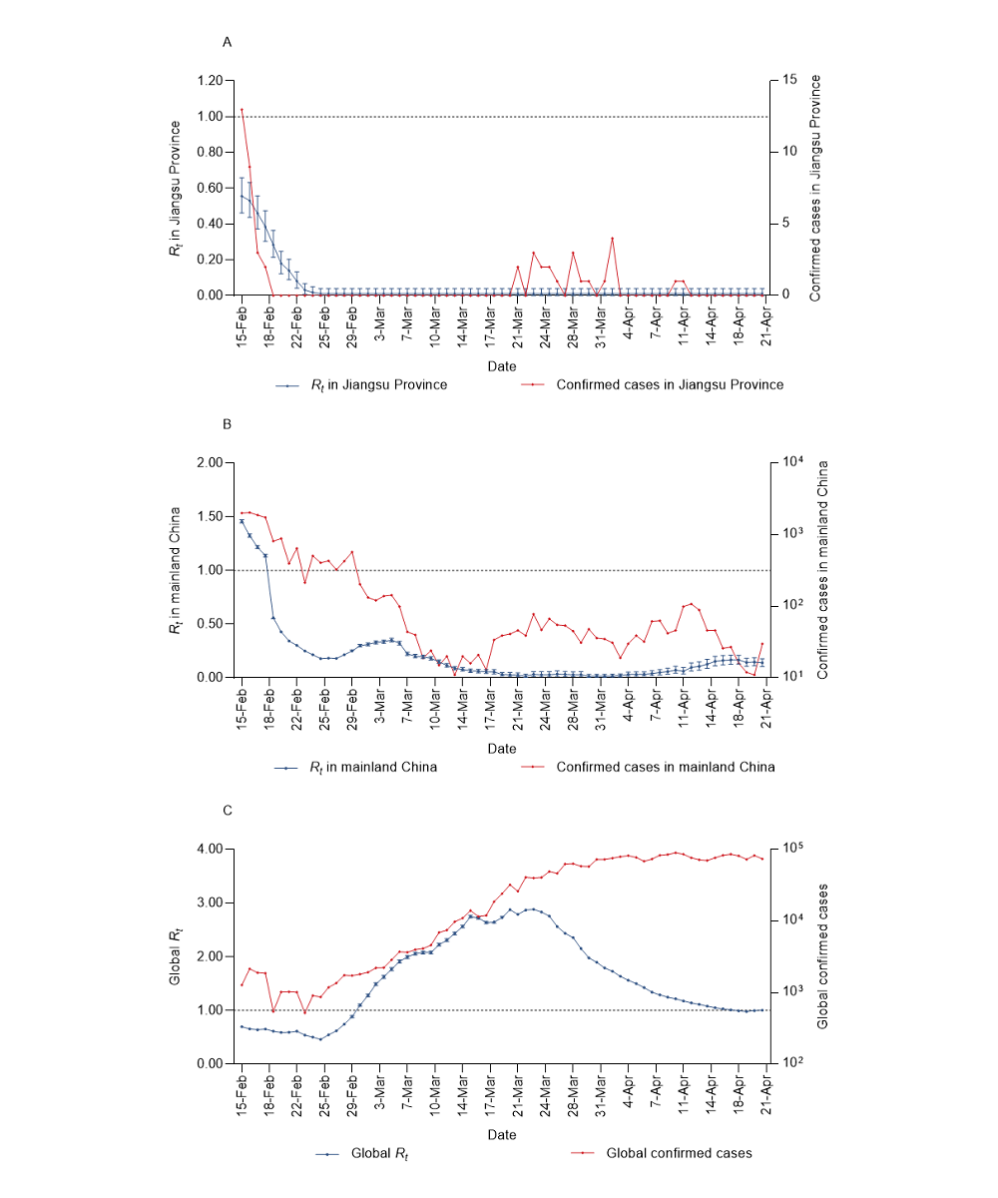
Time-varying reproduction numbers (R_t_), their 95% CIs, and confirmed cases for Jiangsu Province, mainland China, and the entire world.

**Figure 4:**
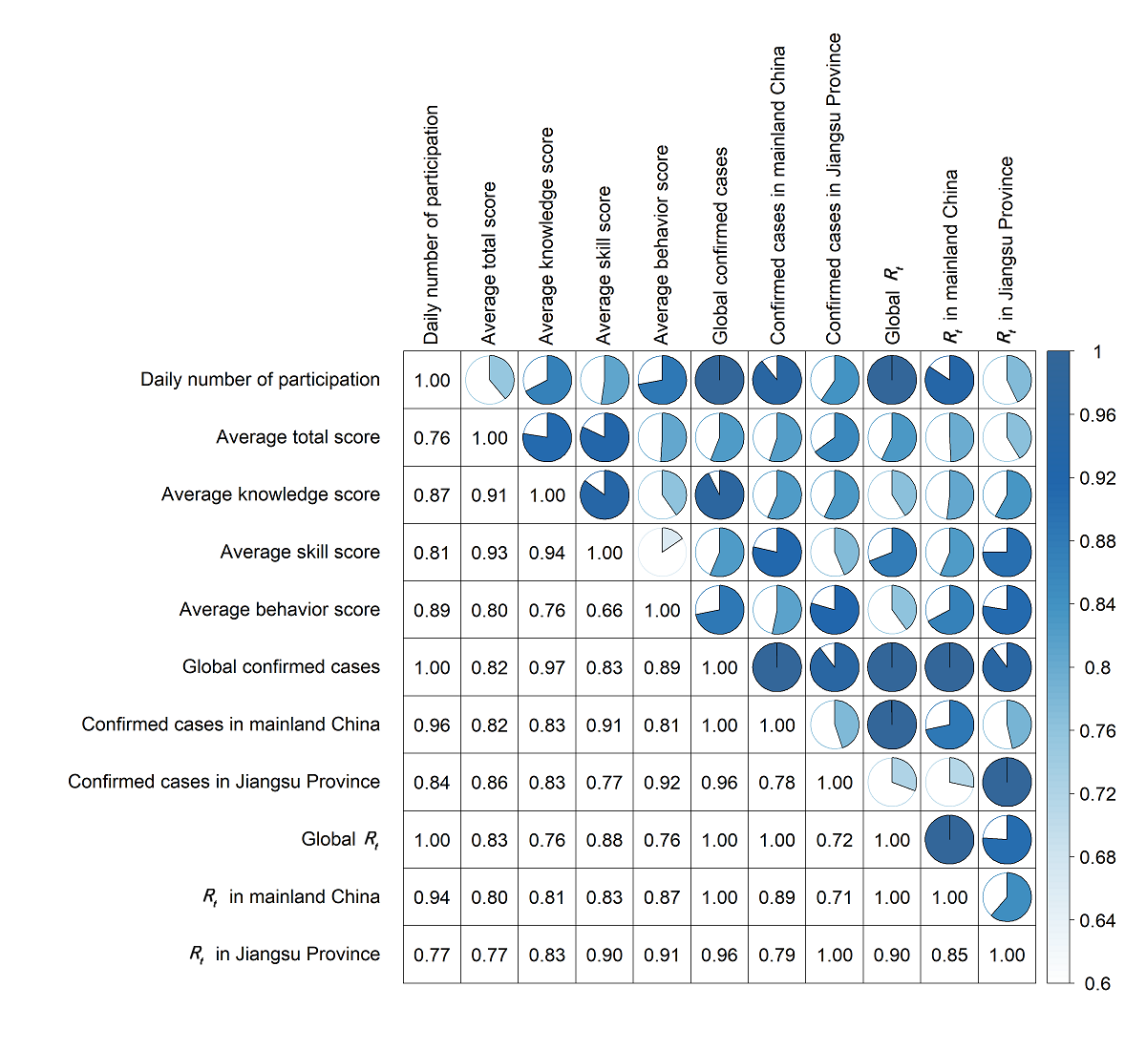
Correlation results for the daily participation number, average protection score, confirmed cases, and time-varying reproduction number (R_t_).

We analyzed the *R_t_* trends and attempted to determine associations among the daily participation number, average protection score, number of confirmed cases, and *R_t_* values during the investigation. Owing to the implementation of strict control measures [21], the *R_t_* in Jiangsu Province declined below 1, close to 0, and the *R_t_* in mainland China also dropped significantly (Figures 3A and B). However, the number of confirmed cases worldwide has been increasing, with the global *R_t_* showing a trend of first rising and then declining and maintaining a value around 1 (Figure 3C). The correlation analysis revealed that the daily participation number and average protection score were significantly associated with the number of confirmed cases and *R_t_* in Jiangsu Province, mainland China, and the entire world (maximal information coefficient >0.70, range: 0.76-1.00; Figure 4).

## Discussion

### Principal Results and Comparison With Prior Work

Emerging infectious diseases are usually unpredictable with a lack of effective vaccines and drug treatments, and have direct or indirect negative impacts on economic development, social stability, and the public’s quality of life [22,23]. During the prior outbreaks of SARS and MERS, investigations were conducted to understand the public’s knowledge, attitudes, and practices [24-29]. Similarly, this study has shown that there was a relatively strong relationship between epidemic development and public perceptions.

A total of 52,066 participants were included in the study, of whom 65.91% had a total protection score above 90 points, indicating that the protection knowledge and skills were well understood and the actual action ability was strong. Unfortunately, there were still deficiencies in knowledge, skills, and actual behaviors in 34.09% of participants, and, hence, precise health education measures need to be provided.

For the knowledge section, the correct answer rates for initial symptoms, transmission routes, and selection of disinfection products were less than 80%; specifically, the rate for initial symptoms was only 62.01% (32,286/52,066), which was less than that of hospital visitors [14]. This may be because the outbreak time of COVID-19 overlaps with that of the common cold, flu, and other diseases, and these are also respiratory diseases, which have certain similarities in clinical manifestations. Similarly, owing to limited knowledge on COVID-19, which is currently an emerging infectious disease, individuals will tend to choose transmission routes and disinfection products that they consider reasonable. It is suggested that relevant departments should release authoritative health information on COVID-19 in a timely manner and strengthen the promotion and education of daily protection knowledge to meet the public’s needs.

For the skill section, the correct rate for home quarantine measures was relatively low; 23.10% (12,029/52,066) of the respondents were unaware that they cannot participate in family dinners. Yet, it is necessary to ensure that dishes and chopsticks are used and sterilized separately to avoid cross-infection. This may be attributed to the fact that although most quarantined individuals have no symptoms or mild symptoms, there is still a probability of presymptomatic transmission [30], which prompts relevant departments to provide health tips on epidemic prevention and control and consider such families as key monitoring objects.

For the actual behavior section, most individuals could reduce risk behaviors and take necessary protective measures, similar with previous study findings [11,13,31]. However, the proportion of those able to disinfect their homes weekly and distinguish the initial symptoms between the common cold and COVID-19 was lower than that of those presenting other behaviors, as in a previous study [14]. This may be related to the limited knowledge on COVID-19 and the lack of self-protection ability, indicating that public health information literacy needs to be improved. In the regression analysis, those with high educational levels were relatively unable to disinfect their homes weekly and clearly distinguish between the common cold and COVID-19. The reason may be that these individuals usually pay more attention to personal protection and that they are more cautious in answering the questions on the difference between the common cold and COVID-19.

Sex, age, educational level, occupation, and place of residence affected the total protection score for COVID-19 at different degrees. Women (average score of 90.58, SD 8.66) tended to have higher total protection scores than men, which is similar to the findings of previous investigations [13,31]. A large difference between men and women was observed in the skill scores—23.88 (SD 4.14) for men and 24.27 (SD 3.84) for women. Conversely, the total protection scores of those aged 21-60 years tended to be higher than the scores of those aged ≤20 years, which may be attributed to the current situation of resuming work and study; thus, these individuals need to actively obtain information on protection knowledge for COVID-19 and improve their self-protection ability. The total score was influenced by the educational level; the total score of those with higher educational levels tended to be higher than that of those with lower educational levels. Relevant studies also showed that individuals with higher educational levels were more willing to accept new knowledge and skills and adopt healthier practices [14,32,33]. In comparison with the unemployed, all participants, except the retirees and especially those with occupations categorized under government agency and institution, enterprise, business and service industry, medical practitioners, and students were more likely to have higher total scores, which may be attributed to their professional characteristics. Those living in urban areas had higher total protection scores than those living in rural areas. This may be attributed to the insufficient basic medical resources and relatively weak primary public health prevention strategies in rural areas. Further, the results may be associated with the relatively limited access to the internet and online health information resources [13]. Therefore, it is necessary to focus on the dissemination of health education knowledge on COVID-19 for men, those aged ≤20 years, those with an educational level of junior high school or less, those who are unemployed, and those living in rural areas.

This study showed that the government, television shows, and news outlets were the main sources for protection knowledge, which accounted for a higher proportion than that in a US sample [12]. Information released by the government and authoritative medical experts was also considered as reliable information, which is different from that reported in a previous study [31]. Moreover, this study emphasizes the need to continue to publicize the latest epidemic developments, disease treatment progress, daily protection knowledge, and other information on COVID-19.

### Limitations

There were several limitations in this study. First, the questionnaire used was designed based on a literature review and was used for the investigation after being revised via expert consultations. With the absence of a rigorous design process, the reliability of information may decline. Second, since the online surveys were conducted through WeChat and only in Jiangsu Province, the research samples are biased and limited. Given the differences in the resumption of work and study across different regions, our conclusions may change when expanding the study population. Lastly, the questionnaire only considered the influencing factors of the total protection score for COVID-19 from an individual perspective, without considering the influence of macro factors, such as government policies and society.

### Conclusion

A high proportion of study participants had good protection knowledge and skills related to COVID-19. The factors influencing the total protection score for COVID-19 included sex, age, educational level, occupation, and place of residence. The study results suggest that relevant government departments need to update accurate information on COVID-19 in a timely manner, such as the latest epidemic developments and disease treatment progress, via official media and new media channels, and continue to promote daily protection knowledge on COVID-19. When resuming work and study, relevant departments need to apply different health education measures and conduct extensive and in-depth health education and promotion activities to guide the public, especially men, younger individuals, individuals with low educational levels, the unemployed, and individuals living in rural areas, to adopt positive and healthy behaviors. Doing so will ultimately reduce the negative impact of COVID-19.

## Appendix

Multimedia Appendix 1 Questionnaire.

Multimedia Appendix 2 Supplementary tables.

## Abbreviations

BIP-Q5: Brief Illness Perception Questionnaire
Jiangsu Provincial CDC: Jiangsu Provincial Center for Disease Control and Prevention
MERS: Middle East respiratory syndrome
R_t_: time-varying reproduction number
SARS: severe acute respiratory syndrome
WHO: World Health Organization
