# Supplementary material for: Chinese Residents’ Perceptions of COVID-19 During the Pandemic: Online Cross-sectional Survey Study
Source: J Med Internet Res. 2020 Nov 25;22(11):e21672. doi: 10.2196/21672 (PMC7690970; doi:10.2196/21672)
Supplement: Multimedia Appendix 1 [file jmir_v22i11e21672_app1.pdf]

Multimedia Appendix 1: Survey questionnaire

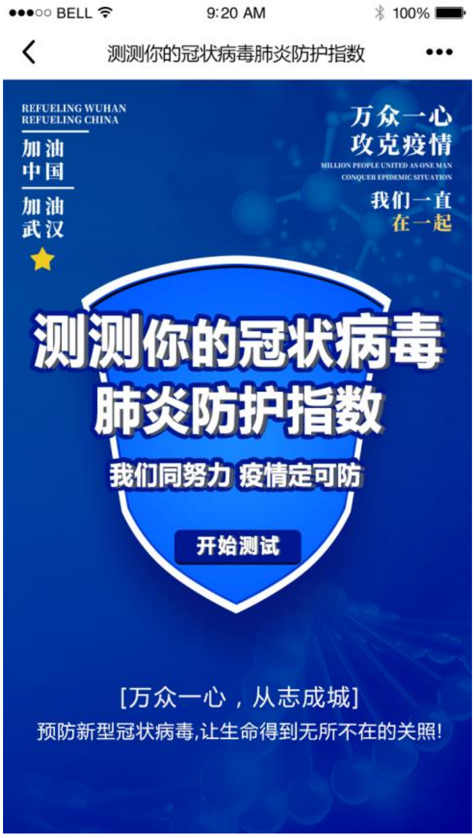

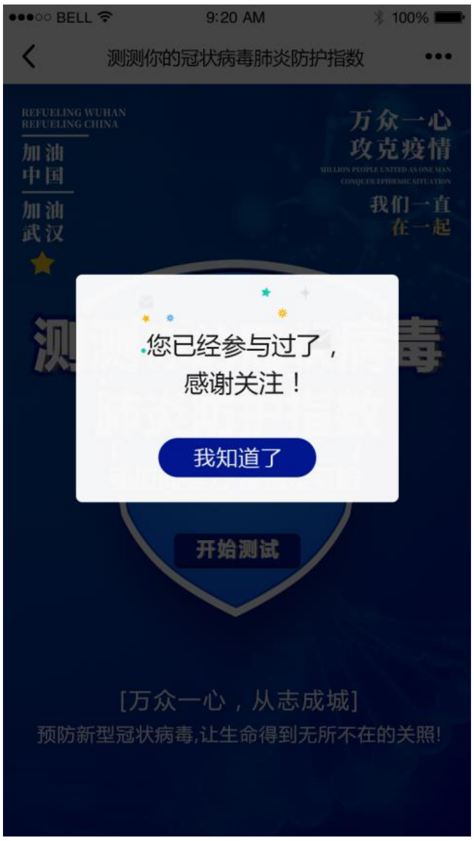

The left picture above is the front page of the survey questionnaire, and the right figure shows that the mobile phone holder has filled in the questionnaire.

Knowledge section

1. Initial symptoms:

A fever, cough, fatigue　　B nausea, vomiting, diarrhea

C panic, chest tightness, conjunctivitis　　D all

2. Distribution of death cases:

A infant　　B middle-aged

C the elderly and those with chronic basic diseases　　D teenagers

3. Not the transmission routes:

A droplet spread　　B mosquito bites

C contact spread　　D all

4. Conditions for killing viruses:

A at 56 ℃ for 30 minutes　　B at 26 ℃ for 20 minutes

C at 16 ℃ for 10 minutes　　D at 0 ℃ for 5 minutes

5. Unsuitable mask selection:

A paper mask　　B disposable medical mask

C medical surgical mask　　D N95 protective mask

6. Medical observation days:

A 7 days　　B 8 days　　C 14 days　　D 30 days

7. Fever temperature:
A 38℃ B 38.5℃ C 37.3℃ D 36.5℃
8. Possible COVID-19 infection after influenza vaccination:
A right B wrong C unclear
9. Selection of disinfection products:
A 75% alcohol B chlorine-containing disinfectant (such as 84 disinfectant)
C wash-free disinfectant D all
10. Infectiousness of asymptomatic people:
A right B wrong C unclear

Skill section
1. When coughing or sneezing, the correct treatment:
A cover your mouth and nose directly with your hand
B cover your mouth and nose with a handkerchief, paper towel or elbow
C no need to cover your mouth and nose
D unclear
2. Incorrect measures of home quarantine:
A wear a mask, and try to keep more than one meter away from your family
B participate in family meals, and share dishes and chopsticks
C clean all objects and surfaces in high frequency contact every day
D pay attention to drinking more water during quarantine
3. Incorrect measures from outside to inside:
A take off the outer clothes, and replace them with household clothes
B after removing the mask, throw it into the garbage can and do not place it at will
C wash hands after handling masks and clothes
D close the window to prevent the outside air from entering
4. Incorrect mask use:
A it is recommended to replace every 2-4 hours
B replace it as soon as possible in case of pollution
C avoid hand contact with the inner face of the mask when wearing
D the thicker the mask, the better the antivirus effect
5. Return notice:
A carry disinfectant wipes with you B do not touch the outer surface of the mask
C avoid touching eyes, mouth and nose with hands D all
6. Washing hands correctly:
A flowing clear water B rub soap or hand sanitizer thoroughly
C duration is more than 15-20 seconds D all
7. Precautions:
A wash hands before eating B avoid going to crowded places
C maintain indoor air circulation D all
8. When there is fever during the travel by public transportation, where should I get off and isolate immediately:
A destination B get off nearby C do not get off D departure station
9. Attention to household alcohol disinfection:

A alcohol is a flammable substance, and should be used carefully
B can be used for hand and skin and small-scale object surface
C generally use 75% concentration
D all

Behavior section (0-3: "be unable to do" to "be able to do")

Behavior	Actual degree
1.No partying	
2.Wearing masks	
3.Wearing gloves	
4.Washing hands	
5.No contact with live poultry	
6.Daily ventilation	
7.Weekly disinfection	
8.Distinction between common cold and COVID-19	
9.Correct identification of epidemic information	
10.Workplace precautions	
11.Community precautions	

Transmission section

1. Information channels to learn protection knowledge:
A government's WeChat public account
B information from WeChat and WeChat group of friends
C microblog
D TV, government's websites and news outlets
E communication between people around and relatives
F others
2. Reliable information source of protection knowledge:
A information released by government's media and WeChat public account
B information released by authoritative medical experts
C information from WeChat and WeChat group of friends
D information from people around and relatives
E others
3. Information needs:
A daily protection knowledge
B latest epidemic development
C disease treatment progress
D current status of epidemic area prevention and control
E material supply
F social dynamics
G others

Personal characteristics

1. Sex:

A male B female

2. Age (years):

A ≤20 B 21-30 C 31-40 D 41-50 E 51-60 F ≥61

3. Education level:

A junior high school and below B high school and technical secondary school

C junior college and Bachelor D master and above

4. Occupation:

A government agency and institution B medical practitioner

C enterprise D business and service industry

E farmer (including agriculture, forestry, animal husbandry, sideline occupations and fishery)

F student G freelancer

H retiree I unemployed

J others

5. Place of residence:

A urban area B rural area
