# Supplementary material for: Chinese Residents’ Perceptions of COVID-19 During the Pandemic: Online Cross-sectional Survey Study
Source: J Med Internet Res. 2020 Nov 25;22(11):e21672. doi: 10.2196/21672 (PMC7690970; doi:10.2196/21672)
Supplement: Multimedia Appendix 2 [file jmir_v22i11e21672_app2.pdf]

Multimedia Appendix 2: supplementary tables

Content

Table S1. Results of multivariate linear regression analysis on knowledge score, skill score and behavior score. 2

Table S2. Pearson Chi-square test for questions with high error rates in knowledge and skill sections, and questions with low proportion of "3 points" in behavior section.......... 6

Table S3. Results of binary logistic regression analysis on factors influencing the choice for initial symptoms, transmission routes, disinfection products, measures of home quarantine, weekly disinfection, and distinction between common cold and COVID-19.......... 8

Table S4. Proportion of information channels and reliable information sources for different characteristics (%).......... 11

Table S5. Proportion of information needs for different characteristics (%).......... 13

Table S1. Results of multivariate linear regression analysis on knowledge score, skill score and behavior score.

Variable	Coefficient	SE	95% CI	*t* test	*P* value	Collinearity statistics (VIF[a])
Model 1: Knowledge score, F test (19,52046)=276.042, P<.001						
Constant	22.045	0.149	21.752 to 22.338	147.512	<.001	
**Sex (Reference group=Male)**						
Female	0.353	0.037	0.281 to 0.426	9.513	<.001	1.082
**Age (years, Reference group=≤20)**						
21-30	1.542	0.106	1.334 to 1.751	14.506	<.001	7.224
31-40	1.752	0.109	1.539 to 1.965	16.133	<.001	8.821
41-50	1.474	0.112	1.255 to 1.694	13.193	<.001	6.183
51-60	0.831	0.129	0.579 to 1.083	6.461	<.001	2.919
≥61	-0.299	0.257	-0.804 to 0.205	-1.162	=.245	1.467
**Educational level (Reference group=≤Junior high school)**						
High school and technical secondary school	0.845	0.050	0.748 to 0.942	17.017	<.001	1.515
Junior college and bachelor	1.885	0.051	1.786 to 1.984	37.312	<.001	1.985
≥Master	2.146	0.095	1.960 to 2.332	22.613	<.001	1.296
**Occupation (Reference group=Unemployed)**						
Government agency and institution	0.926	0.131	0.669 to 1.182	7.071	<.001	3.531
Medical practitioner	1.830	0.135	1.565 to 2.096	13.523	<.001	2.872
Enterprise	0.683	0.114	0.459 to 0.907	5.987	<.001	9.525
Business and service industry	0.326	0.121	0.090 to 0.563	2.706	=.007	4.710
Farmer[b]	0.250	0.138	-0.021 to 0.521	1.810	=.070	2.598
Student	1.098	0.146	0.812 to 1.385	7.516	<.001	6.500
Freelancer	0.111	0.120	-0.123 to 0.345	0.930	=.352	4.879
Retiree	-0.021	0.247	-0.504 to 0.463	-0.083	=.934	1.569
Others	0.131	0.120	-0.105 to 0.367	1.089	=.276	4.613

**Place of residence (Reference group=Rural area)**						
Urban area	0.478	0.040	0.400 to 0.556	11.981	<.001	1.146
Model 2: Skill score, F test (19,52046)=278.829, P<.001						
Constant	19.936	0.142	19.657 to 20.215	140.158	<.001	
**Sex (Reference group=Male)**						
Female	0.439	0.035	0.370 to 0.509	12.428	<.001	1.082
**Age (years, Reference group=≤20)**						
21-30	2.123	0.101	1.924 to 2.321	20.976	<.001	7.224
31-40	2.401	0.103	2.198 to 2.603	23.227	<.001	8.821
41-50	2.295	0.106	2.087 to 2.504	21.578	<.001	6.183
51-60	1.570	0.122	1.330 to 1.810	12.825	<.001	2.919
≥61	0.719	0.245	0.239 to 1.199	2.935	=.003	1.467
**Educational level (Reference group=≤Junior high school)**						
High school and technical secondary school	0.910	0.047	0.817 to 1.002	19.252	<.001	1.515
Junior college and bachelor	1.789	0.048	1.694 to 1.883	37.203	<.001	1.985
≥Master	1.765	0.090	1.588 to 1.942	19.534	<.001	1.296
**Occupation (Reference group=Unemployed)**						
Government agency and institution	0.760	0.125	0.516 to 1.004	6.098	<.001	3.531
Medical practitioner	1.250	0.129	0.998 to 1.503	9.707	<.001	2.872
Enterprise	0.801	0.109	0.588 to 1.014	7.378	<.001	9.525
Business and service industry	0.568	0.115	0.343 to 0.793	4.947	<.001	4.710
Farmer[b]	0.334	0.131	0.076 to 0.592	2.540	=.011	2.598
Student	1.336	0.139	1.064 to 1.609	9.607	<.001	6.500
Freelancer	0.233	0.114	0.010 to 0.456	2.048	=.041	4.879
Retiree	-0.147	0.235	-0.608 to 0.313	-0.627	=.531	1.569
Others	0.231	0.114	0.006 to 0.455	2.014	=.044	4.613

**Place of residence (Reference group=Rural area)**						
Urban area	0.323	0.038	0.248 to 0.397	8.504	<.001	1.146
Model 3: Behavior score, F test (19,52046)=23.486, P<.001						
Constant	30.118	0.105	29.912 to 30.325	286.196	<.001	
**Sex (Reference group=Male)**						
Female	0.067	0.026	0.016 to 0.118	2.555	=.011	1.082
**Age (years, Reference group=≤20)**						
21-30	0.459	0.075	0.313 to 0.606	6.135	<.001	7.224
31-40	0.769	0.076	0.619 to 0.919	10.055	<.001	8.821
41-50	0.681	0.079	0.527 to 0.836	8.658	<.001	6.183
51-60	0.516	0.091	0.339 to 0.694	5.701	<.001	2.919
≥61	-0.212	0.181	-0.567 to 0.144	-1.168	=.243	1.467
**Educational level (Reference group=≤Junior high school)**						
High school and technical secondary school	0.207	0.035	0.139 to 0.276	5.924	<.001	1.515
Junior college and bachelor	0.071	0.036	0.001 to 0.141	1.991	=.046	1.985
≥Master	-0.096	0.067	-0.227 to 0.035	-1.430	=.153	1.296
**Occupation (Reference group=Unemployed)**						
Government agency and institution	0.726	0.092	0.546 to 0.907	7.880	<.001	3.531
Medical practitioner	0.978	0.095	0.791 to 1.165	10.264	<.001	2.872
Enterprise	0.529	0.080	0.371 to 0.686	6.583	<.001	9.525
Business and service industry	0.683	0.085	0.516 to 0.849	8.039	<.001	4.710
Farmer[b]	0.444	0.097	0.253 to 0.634	4.561	<.001	2.598
Student	0.998	0.103	0.796 to 1.199	9.696	<.001	6.500
Freelancer	0.615	0.084	0.450 to 0.780	7.308	<.001	4.879
Retiree	0.737	0.174	0.396 to 1.078	4.239	<.001	1.569
Others	0.520	0.085	0.354 to 0.686	6.139	<.001	4.613

**Place of residence (Reference group=Rural area)**						
Urban area	0.135	0.028	0.080 to 0.190	4.809	<.001	1.146

[a]VIF: variance inflation factor.

[b]"Farmer" includes agriculture, forestry, animal husbandry, sideline occupations, and fishery.

Table S2. Pearson Chi-square test for questions with high error rates in knowledge and skill sections, and questions with low proportion of "3 points" in behavior section.

Characteristic	Initial symptoms	Transmission routes	Selection of disinfection products	Measures of home quarantine	Weekly disinfection	Distinction between common cold and COVID-19[a]
**Sex**						
Male	χ^2=87.8	χ^2=190.5	χ^2=121.8	χ^2=47.0	χ^2=2.8	χ^2=23.7
Female	*P*<.001	*P*<.001	*P*<.001	*P*<.001	*P*=.429	*P*<.001
**Age (years)**						
≤20						
21-30						
31-40	χ^2=244.4	χ^2=1,005.0	χ^2=363.2	χ^2=534.6	χ^2=432.9	χ^2=291.4
41-50	*P*<.001	*P*<.001	*P*<.001	*P*<.001	*P*<.001	*P*<.001
51-60						
≥61						
**Educational level**						
≤Junior high school						
High school and technical secondary school	χ^2=304.3	χ^2=1,257.6	χ^2=1,056.6	χ^2=803.5	χ^2=410.1	χ^2=269.2
Junior college and bachelor	*P*<.001	*P*<.001	*P*<.001	*P*<.001	*P*<.001	*P*<.001
≥Master						
**Occupation**						
Government agency and institution						
Medical practitioner						
Enterprise	χ^2=320.4	χ^2=1,031.6	χ^2=633.3	χ^2=653.1	χ^2=356.8	χ^2=404.3
Business and service industry	*P*<.001	*P*<.001	*P*<.001	*P*<.001	*P*<.001	*P*<.001
Farmer[b]						
Student						
Freelancer						

Retiree						
Unemployed						
Others						
**Place of residence**						
Urban area	χ^2=38.4	χ^2=369.1	χ^2=217.6	χ^2=84.6	χ^2=22.2	χ^2=33.3
Rural area	*P*<.001	*P*<.001	*P*<.001	*P*<.001	*P*<.001	*P*<.001

[a]COVID-19: coronavirus disease 2019.

[b]"Farmer" includes agriculture, forestry, animal husbandry, sideline occupations, and fishery.

Table S3. Results of binary logistic regression analysis on factors influencing the choice for initial symptoms, transmission routes, disinfection products, measures of home quarantine, weekly disinfection, and distinction between common cold and COVID-19.

Variable	Initial symptoms		Transmission routes		Selection of disinfection products		Measures of home quarantine		Weekly disinfection		Distinction between common cold and COVID-19[a]	
	OR[b] (95% CI)	*P* value	*OR* (95% CI)	*P* value	*OR* (95% CI)	*P* value	*OR* (95% CI)	*P* value	*OR* (95% CI)	*P* value	*OR* (95% CI)	*P* value
Constant	N/A[c]	=.009	N/A	<.001	N/A	=.002	N/A	<.001	N/A	<.001	N/A	<.001
**Sex (Reference group=Male)**												
Female	1.133 (1.091 to 1.176)	<.001	1.207 (1.159 to 1.258)	<.001	1.164 (1.117 to 1.212)	<.001	1.114 (1.067 to 1.164)	<.001	N/A	N/A	N/A	N/A
**Age (years, Reference group=≤20)**		<.001		<.001		<.001		<.001		<.001		<.001
21-30	0.979 (0.878 to 1.091)	=.699	1.034 (0.918 to 1.164)	=.582	1.298 (1.154 to 1.459)	<.001	1.642 (1.458 to 1.850)	<.001	1.036 (0.913 to 1.175)	=.584	0.899 (0.787 to 1.027)	=.116
31-40	1.057 (0.947 to 1.181)	=.325	0.949 (0.841 to 1.069)	=.388	1.332 (1.183 to 1.500)	<.001	1.710 (1.515 to 1.929)	<.001	1.423 (1.249 to 1.620)	<.001	1.129 (0.985 to 1.293)	=.081
41-50	0.869 (0.776 to 0.974)	=.016	0.960 (0.848 to 1.086)	=.512	1.081 (0.957 to 1.222)	=.208	1.619 (1.429 to 1.835)	<.001	1.464 (1.279 to 1.675)	<.001	1.164 (1.011 to 1.340)	=.035
51-60	0.718 (0.631 to 0.818)	<.001	0.695 (0.604 to 0.799)	<.001	0.943 (0.821 to 1.084)	=.411	1.296 (1.123 to 1.495)	<.001	1.494 (1.275 to 1.751)	<.001	1.070 (0.910 to 1.259)	=.412
≥61	0.739 (0.573 to 0.953)	=.020	0.420 (0.323 to 0.546)	<.001	0.862 (0.660 to 1.127)	=.279	0.984 (0.749 to 1.294)	=.909	1.158 (0.845 to 1.586)	=.361	1.029 (0.738 to 1.435)	=.866
**Educational level (Reference group=≤Junior high school)**		<.001		<.001		<.001		<.001		<.001		<.001
High school and technical secondary school	1.097 (1.044 to 1.152)	<.001	1.152 (1.094 to 1.213)	<.001	1.200 (1.139 to 1.264)	<.001	1.168 (1.105 to 1.234)	<.001	0.932 (0.874 to 0.994)	=.031	1.165 (1.093 to 1.241)	<.001
Junior college and bachelor	1.298	<.001	1.747	<.001	1.649	<.001	1.459	<.001	0.672	<.001	1.008	=.799

	(1.235 to 1.364)		(1.654 to 1.845)		(1.562 to 1.741)		(1.379 to 1.544)		(0.631 to 0.716)		(0.948 to 1.072)	
≥Master	1.432 (1.299 to 1.578)	<.001	2.235 (1.989 to 2.512)	<.001	1.847 (1.651 to 2.065)	<.001	1.368 (1.222 to 1.531)	<.001	0.517 (0.464 to 0.577)	<.001	0.871 (0.778 to 0.975)	=.017
**Occupation (Reference group=Unemployed)**		<.001		<.001		<.001		<.001		<.001		<.001
Government agency and institution	1.404 (1.230 to 1.603)	<.001	1.302 (1.125 to 1.506)	<.001	1.251 (1.085 to 1.443)	=.002	1.291 (1.110 to 1.501)	=.001	1.459 (1.252 to 1.699)	<.001	1.430 (1.223 to 1.673)	<.001
Medical practitioner	1.583 (1.379 to 1.818)	<.001	1.771 (1.515 to 2.070)	<.001	1.948 (1.668 to 2.275)	<.001	1.946 (1.649 to 2.296)	<.001	2.370 (2.000 to 2.808)	<.001	2.843 (2.367 to 3.414)	<.001
Enterprise	1.080 (0.964 to 1.210)	=.182	1.036 (0.918 to 1.170)	=.565	1.156 (1.025 to 1.305)	=.018	1.239 (1.091 to 1.408)	=.001	1.217 (1.068 to 1.388)	=.003	1.134 (0.994 to 1.294)	=.062
Business and service industry	1.077 (0.956 to 1.215)	=.224	0.952 (0.838 to 1.083)	=.455	1.027 (0.904 to 1.166)	=.683	1.134 (0.991 to 1.298)	=.068	1.430 (1.242 to 1.647)	<.001	1.480 (1.283 to 1.708)	<.001
Farmer[d]	1.123 (0.980 to 1.288)	=.096	0.980 (0.848 to 1.132)	=.779	1.109 (0.959 to 1.281)	=.162	1.082 (0.929 to 1.260)	=.310	1.470 (1.242 to 1.742)	<.001	1.234 (1.048 to 1.454)	=.012
Student	1.311 (1.132 to 1.519)	<.001	1.161 (0.990 to 1.363)	=.067	1.430 (1.220 to 1.675)	<.001	1.346 (1.143 to 1.585)	<.001	1.740 (1.468 to 2.063)	<.001	1.596 (1.338 to 1.902)	<.001
Freelancer	1.031 (0.915 to 1.160)	=.618	0.939 (0.827 to 1.066)	=.330	0.966 (0.852 to 1.095)	=.584	1.033 (0.904 to 1.179)	=.634	1.477 (1.283 to 1.701)	<.001	1.403 (1.218 to 1.616)	<.001
Retiree	0.951 (0.746 to 1.212)	=.684	0.867 (0.673 to 1.117)	=.270	0.907 (0.704 to 1.169)	=.450	0.931 (0.715 to 1.213)	=.596	1.316 (0.969 to 1.787)	=.079	1.471 (1.071 to 2.021)	=.017
Others	1.113 (0.987 to 1.254)	=.080	0.899 (0.792 to 1.021)	=.102	0.995 (0.877 to 1.130)	=.944	0.997 (0.872 to 1.139)	=.961	1.364 (1.185 to 1.569)	<.001	1.233 (1.071 to 1.420)	=.004
**Place of residence (Reference group=Rural area)**												
Urban area	N/A	N/A	1.206 (1.156 to 1.258)	<.001	1.058 (1.013 to 1.104)	=.010	N/A	N/A	1.053 (1.002 to 1.106)	=.042	N/A	N/A

[a]COVID-19: coronavirus disease 2019.

[b]OR: odds ratio.

[c]N/A: not applicable.

[d]"Farmer" includes agriculture, forestry, animal husbandry, sideline occupations, and fishery.

Table S4. Proportion of information channels and reliable information sources for different characteristics (%).

Characteristic	Information channels						Reliable information sources				
	Government's WeChat public account	WeChat and WeChat group of friends	Microblog	TV, government's websites, and news outlets	Communication between people around and relatives	Others	government's media and WeChat public account	authoritative medical experts	WeChat and WeChat group of friends	people around and relatives	Others
**Sex**											
Male	89.15	64.52	47.54	88.67	47.38	30.33	93.93	88.03	7.10	6.63	33.61
Female	85.67	64.81	55.93	88.57	48.41	29.19	91.19	89.07	6.49	5.95	33.76
**Age (years)**											
≤20	76.40	60.38	47.77	79.93	46.04	32.55	84.50	82.23	10.55	9.68	33.85
21-30	87.53	65.57	61.18	87.97	49.74	29.26	93.89	89.93	6.71	6.26	32.15
31-40	89.56	66.40	49.77	90.93	48.21	28.66	94.02	89.74	6.43	5.86	33.43
41-50	90.60	63.84	45.09	90.30	46.19	31.31	93.51	88.74	6.16	5.81	35.94
51-60	88.12	60.84	39.72	88.25	45.43	30.58	93.30	84.96	5.54	5.48	34.74
≥61	81.44	52.35	28.81	80.89	41.27	29.64	83.66	79.22	8.03	7.48	33.24
**Educational level**											
≤Junior high school	81.52	54.58	40.77	84.24	38.64	25.12	87.92	84.42	7.06	6.55	33.38
High school and technical secondary school	87.88	63.42	48.62	89.57	46.38	27.94	93.77	88.89	7.30	6.79	35.10
Junior college and bachelor	91.66	71.42	59.12	91.21	54.48	34.28	95.72	91.04	6.40	5.90	33.65
≥Master	89.76	73.78	57.15	87.87	53.67	30.64	91.48	88.53	6.92	6.47	27.82
**Occupation**											
Government agency and institution	93.77	74.85	58.01	90.56	55.97	34.09	95.33	90.84	8.10	7.54	30.46
Medical practitioner	93.31	76.22	66.31	91.96	63.46	46.52	94.65	93.72	10.81	9.42	44.43
Enterprise	91.03	65.55	50.35	90.94	48.11	29.24	95.28	90.21	4.94	4.86	33.19

Business and service industry	87.79	64.07	51.73	89.33	46.78	25.67	93.96	88.45	6.79	6.30	33.85
Farmer[a]	85.21	58.13	44.03	86.85	41.17	23.17	89.93	84.34	9.59	8.20	32.45
Student	78.79	63.54	52.61	82.35	49.50	32.38	87.03	84.47	10.48	9.13	33.28
Freelancer	84.99	60.17	47.50	87.79	42.65	24.23	91.15	86.96	6.86	6.27	32.01
Retiree	82.86	54.76	33.10	85.71	40.48	25.71	88.81	84.52	7.14	6.90	32.14
Unemployed	81.33	59.73	45.99	85.69	40.77	27.40	89.41	86.19	6.08	6.08	32.26
Others	85.04	61.09	49.00	86.43	44.54	32.97	90.75	87.05	5.87	5.63	35.16
**Place of residence**											
Urban area	89.10	66.15	52.28	89.35	49.22	30.04	93.53	89.22	6.35	5.98	33.02
Rural area	84.95	61.68	48.68	87.22	45.06	29.48	91.33	87.00	7.82	7.05	34.96
**Total**	87.69	64.64	51.06	88.63	47.81	29.85	92.78	88.47	6.85	6.35	33.67

[a]"Farmer" includes agriculture, forestry, animal husbandry, sideline occupations, and fishery.

Table S5. Proportion of information needs for different characteristics (%).

Characteristic	Daily protection knowledge	Latest epidemic development	Disease treatment progress	Current status of epidemic area prevention and control	Material supply	Social dynamics	Others
**Sex**							
Male	79.29	90.04	80.74	75.23	63.77	63.46	18.91
Female	80.06	89.35	81.40	73.59	63.96	59.71	17.58
**Age (years)**							
≤20	78.57	82.58	74.58	66.27	53.90	49.91	21.17
21-30	77.14	90.09	84.23	74.05	66.42	62.98	17.62
31-40	80.49	90.88	82.96	76.85	66.14	64.52	17.13
41-50	81.68	91.04	79.01	75.99	63.12	62.49	19.43
51-60	81.33	90.33	73.71	73.38	59.58	60.44	20.42
≥61	75.35	84.21	64.54	65.65	46.81	55.12	22.99
**Educational level**							
≤Junior high school	79.32	85.07	75.53	70.42	56.16	53.88	16.77
High school and technical secondary school	80.59	90.87	81.59	76.91	63.72	63.93	16.97
Junior college and bachelor	79.81	92.35	84.64	76.45	69.08	66.66	20.55
≥Master	74.31	89.59	79.84	70.18	66.04	58.17	16.43
**Occupation**							
Government agency and institution	81.75	91.65	82.29	75.44	68.04	62.25	20.23
Medical practitioner	85.08	94.09	87.14	82.46	73.82	71.88	28.95
Enterprise	80.28	91.46	83.05	76.41	66.67	65.05	17.27
Business and service industry	78.48	90.79	82.26	75.36	65.00	63.56	16.32
Farmer[a]	79.78	84.56	76.49	73.58	58.39	54.27	14.79
Student	80.17	85.02	77.28	68.53	56.89	53.39	20.99

Freelancer	77.33	89.33	78.19	72.72	60.08	59.65	14.71
Retiree	78.81	90.48	69.76	69.29	55.24	58.10	19.05
Unemployed	75.68	87.55	77.75	71.39	59.23	58.37	15.16
Others	77.70	87.65	79.77	72.83	61.24	60.11	21.35
**Place of residence**							
Urban area	79.58	90.65	81.81	74.89	65.32	62.62	18.05
Rural area	79.67	88.00	79.46	73.86	60.99	60.45	18.95
**Total**	79.61	89.75	81.01	74.54	63.85	61.89	18.35

[a]"Farmer" includes agriculture, forestry, animal husbandry, sideline occupations, and fishery.
